# Evaluation of both perfusion and atrophy in multiple system atrophy of the cerebellar type using brain SPECT alone

**DOI:** 10.1186/1471-2342-10-17

**Published:** 2010-08-11

**Authors:** Hiroshi Matsuda, Etsuko Imabayashi, Ichiei Kuji, Akira Seto, Kimiteru Ito, Daisuke Kikuta, Minoru Yamada, Yasumasa Shimano, Noriko Sato

**Affiliations:** 1 Department of Nuclear Medicine, Saitama Medical University International Medical Center, 1397-1, Yamane, Hidaka, Saitama, 350-1298, Japan; 2 Department of Nuclear Medicine, Saitama Medical University Hospital, 38, Morohongo, Moroyama-machi, Iruma-gun, Saitama,350-0495, Japan; 3 Department of Radiology, National Center Hospital of Neurology and Psychiatry, 4-1-1, Ogawahigashi, Kodaira, Tokyo, 187-8551, Japan

## Abstract

**Background:**

Partial volume effects in atrophied areas should be taken into account when interpreting brain perfusion single photon emission computed tomography (SPECT) images of neurodegenerative diseases. To evaluate both perfusion and atrophy using brain SPECT alone, we developed a new technique applying tensor-based morphometry (TBM) to SPECT.

**Methods:**

After linear spatial normalization of brain perfusion SPECT using ^99m^Tc-ethyl cysteinate dimer (^99m^Tc-ECD) to a Talairach space, high-dimension-warping was done using an original ^99m^Tc-ECD template. Contraction map images calculated from Jacobian determinants and spatially normalized SPECT images using this high-dimension-warping were compared using statistical parametric mapping (SPM2) between two groups of 16 multiple system atrophy of the cerebellar type (MSA-C) patients and 73 age-matched normal controls. This comparison was also performed in conventionally warped SPECT images.

**Results:**

SPM2 demonstrated statistically significant contraction indicating local atrophy and decreased perfusion in the whole cerebellum and pons of MSA-C patients as compared to normal controls. Higher significance for decreased perfusion in these areas was obtained in high-dimension-warping than in conventional warping, possibly due to sufficient spatial normalization to a ^99m^Tc-ECD template in high-dimensional warping of severely atrophied cerebellum and pons. In the present high-dimension-warping, modification of tracer activity remained within 3% of the original tracer distribution.

**Conclusions:**

The present new technique applying TBM to brain SPECT provides information on both perfusion and atrophy at the same time thereby enhancing the role of brain perfusion SPECT

## Background

Brain perfusion single photon emission computed tomography (SPECT) has been applied to various neurodegenerative diseases such as Alzheimer's disease. Particular attention must be paid to local atrophy when interpreting brain SPECT images in neurodegenerative diseases, since tracer activity determined by SPECT with limited spatial resolution is greatly influenced by partial volume effects. Local atrophy causes underestimation of tracer activity due to these effects. To precisely evaluate this local atrophy of the brain, magnetic resonance imaging (MRI) has been commonly used. If SPECT could evaluate local atrophy as well as brain perfusion, the role of SPECT would be enhanced in clinical use. In the present study, we developed a new technique to evaluate both perfusion and atrophic changes at the same time using brain SPECT alone, and applied this technique to patients with multiple system atrophy of the cerebellar type (MSA-C) for clinical validation.

## Methods

We retrospectively chose 16 patients (9 men and 7 women age 43-75 years, mean ± SD 58.6 ± 8.8) with a clinical diagnosis of probable MSA-C according to the consensus criteria [1]: cerebellar ataxia with additional severe autonomic failure and/or a levodopa-unresponsive or a poorly responsive parkinsonian syndrome. All patients showed characteristic brain MRI findings of cerebellar and pontine atrophy and signal changes in the pons [1,2]. Seventy-three age-matched control subjects (35 men and 38 women; age 43-75 years, mean ± SD 59.6 ± 7.8 years) with no memory impairment or cognitive disorders were recruited as healthy volunteers. The Ethics Committee of the National Center of Neurology and Psychiatry approved this study, and all patients and control subjects gave their informed consent to take part in this study and for their images to be used in a scientific publication. All of the control subjects were right handed and screened by questionnaire and medical history to exclude any with medical conditions potentially affecting the central nervous system. In addition, none of them had asymptomatic cerebral infarction detected by T2-weighted MRI.

All of the subjects underwent brain perfusion SPECT. Before SPECT was performed, an intravenous line was established. Each received a 600 MBq intravenous injection of ^99m^Tc-ethyl cysteinate dimer (^99m^Tc-ECD) while lying in the supine position with the eyes closed in a dimly lit, quiet room. Ten minutes after the injection of ^99m^Tc-ECD, brain SPECT was performed using cameras equipped with high-resolution fanbeam collimators (Multispect3; Siemens Medical Systems, Hoffman Estates, IL). A Shepp and Logan Hanning filter was used as a filtered back-projection method for SPECT image reconstruction at 0.7 cycles/cm. Attenuation correction was performed using Chang's method with an optimized effective attenuation coefficient of 0.09 cm^-1^.

**Figure 1:**
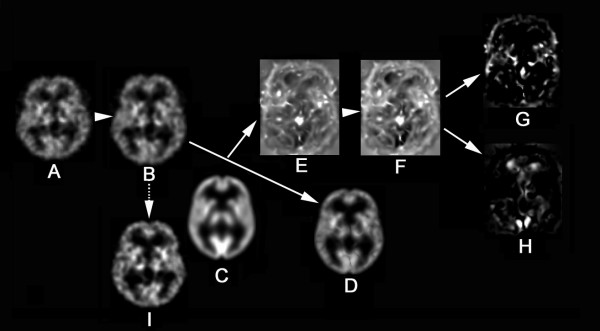
**Procedure of tensor-based morphometry using brain SPECT**. First, an original SPECT image (A) is linearly transformed to the Talairach space. Subsequent non-linear spatial normalization using the high-dimension-warping algorithm deforms a linearly standardized brain (B) to match it to a ^99m^Tc-ECD template (C). This step generates a high-dimensionally warped SPECT image (D) and a parametric image of Jacobian determinants (E) indicating a local volume change relative to the template brain. Then the natural logarithm of this parametric image (F) is separated into negative log J (G) and positive log J maps (H) representing contraction and expansion respectively. A conventionally warped SPECT image is also shown (I).

The present study applied tensor-based morphometry (TBM) to SPECT for the assessment of local atrophy according to the procedure shown in Fig. 1. The basic principle of TBM is to analyze the local deformations of an image and to infer local differences in brain structure [3]. First, a linear spatial normalization algorithm in statistical parametric mapping (SPM2) resized all SPECT images of individual subjects to a voxel size of 2.0 mm and adjusted for orientation, and overall width, length and height. This step transformed brains to the anatomical space of a template brain based on Talairach space. Subsequent non-linear spatial normalization introduced local deformations to each brain to match it to an original template brain for ^99m^Tc-ECD [4]. This non-linear spatial normalization was done using the high-dimension-warping algorithm [5]. We obtained high-dimensionally warped SPECT images and three-dimensional (3D) deformation fields for every brain using a univariate Jacobian approach. Each of these 3 D deformation fields consists of displacement vectors for every voxel, which describes the 3 D displacement needed to locally deform the brain to match it to the template. We calculated the Jacobian determinants to obtain voxel by voxel parametric maps of local volume change relative to the template brain. The local Jacobian determinant is a parameter commonly used in continuum mechanics, which characterizes volume changes, such as local expansion (Jacobian > 1) or contraction (Jacobian < 1) caused by warping. For more fair comparison of regions showing expansion or contraction, we took the natural logarithm of the Jacobian determinant values (denoted as log J) as reported by Leow et al [6]. Then, parametric maps were separated into negative log J and positive log J maps representing contraction and expansion respectively. Absolute values of negative log J were calculated for further analysis of the contraction maps. A greater absolute value of negative log J indicates severer local atrophy. We also spatially normalized all of the perfusion SPECT images to an original ^99m^Tc-ECD template brain using a conventional non-linear warping method with 16 iterations of SPM2.

Contraction map images showing the negative J maps with displacement vectors in each voxel, and high-dimensionally and conventionally warped SPECT images were finally smoothed with a Gaussian kernel with FWHM of 10 mm. For statistical inference, these images were analyzed with a general linear model using SPM2. A design matrix was constructed for two-sample t-test between groups of MSA-C patients and normal controls. Inferences were obtained on t-contrasts. P-values used were corrected with familywise error (threshold at p < 0.05) depending on the contrast.

## Results

**Figure 2:**
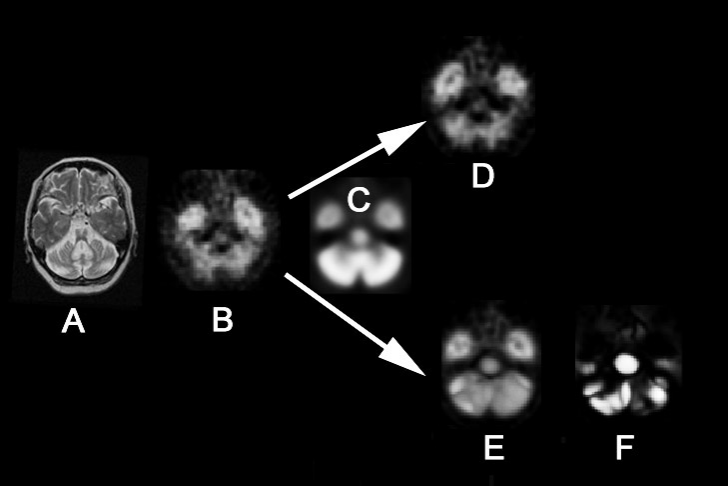
**Conventional warping and high-dimension-warping of a SPECT image in an MSA-C patient**. T2-weighted MRI (A) showed severe atrophy in cerebellum and pons in a 56-year-old woman with MSA-C. A SPECT image at the level of the pons (B) demonstrated perfusion decrease in the cerebellum and pons. In contrast to conventional warping (D), high-dimension-warping (E) achieved sufficient spatial normalization to a template (C) using a contraction map (F).

**Figure 3:**
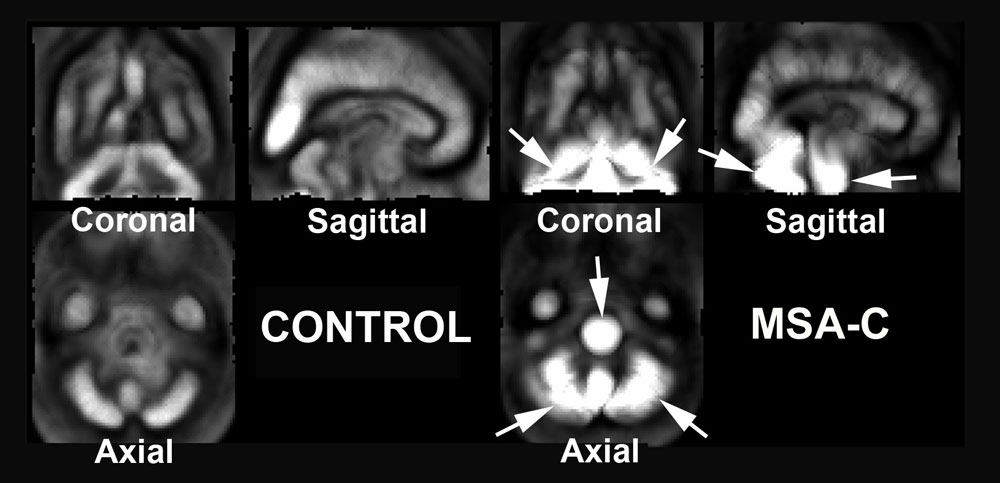
**Averaged images of contraction maps calculated from high-dimension-warping in the groups of normal controls and MSA-C patients**. In the group of MSA-C patients contraction images showed greater absolute values of negative log J indicating severer atrophy in the whole cerebellum and pons than in other areas (arrows). Normal controls did not show severer atrophy in the cerebellum and pons than in other areas.

**Figure 4:**
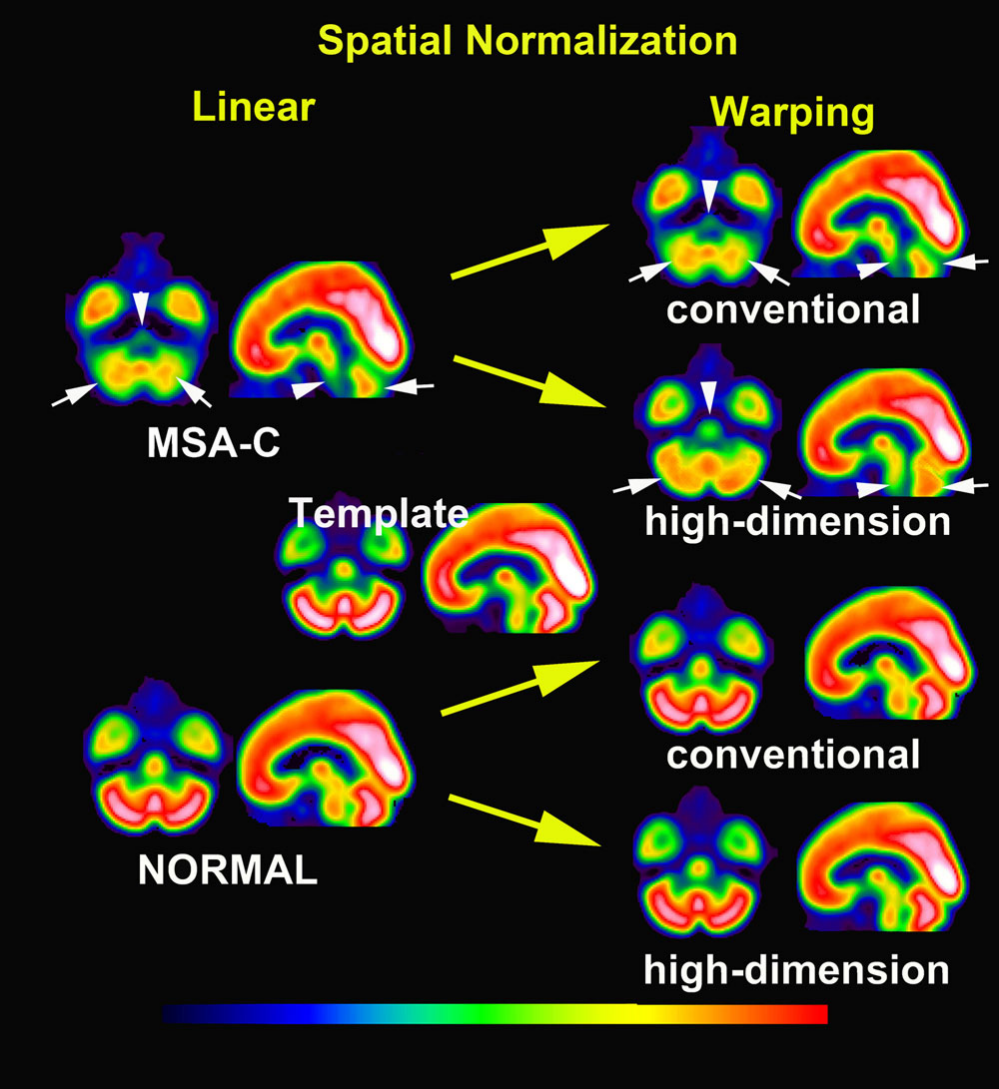
**Averaged images of spatially normalized with linear transformation and subsequent conventionally and high-dimensionally warped SPECT images in groups of MSA-C patients and normal controls**. Linear spatial normalization showed insufficient transformation to a template in cerebellum (white arrows) and pons (white arrowheads) in MSA-C patients. Subsequent conventional warping could not perform a further transformation in these areas in this group. In contrast, high-dimension-warping could fully transform the cerebellum and pons to a template in MSA-C patients. Both conventionally and high-dimensionally warped SPECT images demonstrated decreased perfusion in the cerebellum and pons in MSA-C patients as compared to normal controls.

A contraction map and high-dimensionally and conventionally warped SPECT images in a representative MSA-C patient are shown with the MRI findings in Fig. 2. Averaged images of the contraction maps and high-dimensionally and conventionally warped SPECT images in the groups of MSA-C patients and normal controls are shown in Fig. 3 and Fig. 4. In the group of MSA-C patients the contraction map images showed greater absolute values of negative log J in the whole cerebellum and pons than in other areas (Fig. 3). Both high-dimensionally and conventionally warped SPECT images showed decreased perfusion in these areas in the group of MSA-C patients as compared to the normal controls (Fig. 4). Functional volume of cerebellum and pons in the averaged images was compared between high-dimensional and conventional warping using a region of interest template of WFU_PickAtlas http://www.nitrc.org/projects/wfu_pickatlas/. In the group of MSA-C patients conventional warping generated 16% and 24% smaller volume of cerebellum and pons respectively than high-dimension-warping. Maximal perfusion ratios of the whole cerebellum and pons to the whole cerebrum were calculated in averaged images also using a region of in-terest template of WFU_PickAtlas. Conventionally warped and high-dimensionally warped SPECT images in the group of MSA-C patients showed maximal perfusion ratios of 0.81 and 0.83 in the whole cerebellum and of 0.55 and 0.58 in the pons on the average respectively. Conventionally warped and high-dimensionally warped SPECT images in the group of normal controls showed maximal perfusion ratios of 0.90 and 0.88 in the whole cerebellum and of 0.68 and 0.65 in the pons on average, respectively.

**Figure 5:**
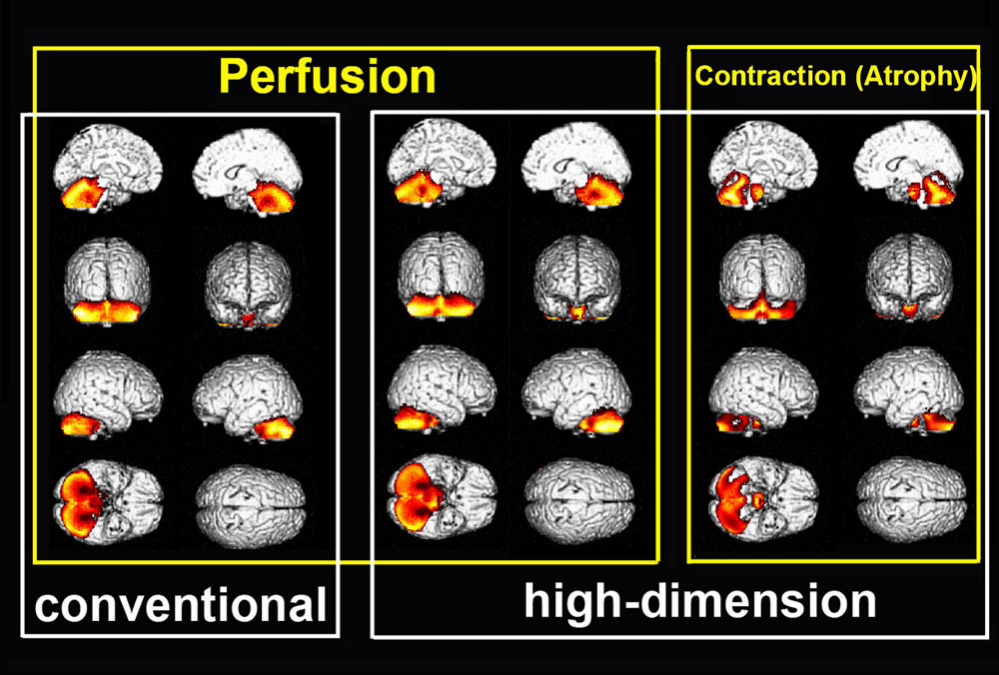
**Group comparison of warped SPECT and contraction images between MSA-C patients and normal controls**. High-dimension-warping demonstrates significantly decreased perfusion and significant contraction indicating local atrophy as a colored map in the whole cerebellum and pons of MSA-C patients as compared to normal controls. Conventional warping also demonstrated significantly decreased perfusion in the same area as in the high-dimension warping.

Group comparisons of these images demonstrated significant contraction indicating local atrophy and decreased perfusion in the whole cerebellum and pons in MSA-C patients as compared to normal controls (Fig. 5 and Table 1). Higher t-values and wider cluster size were obtained with high-dimension-warping as compared to conventional warping.

**Table 1:** Location of significant perfusion decrease and contraction indicating atrophy in MSA-C patients as compared with normal controls

conventional warping (perfusion)					
		Talairach coordinate			
			
Cluster size	t value	x	y	z	Region
22172	19.9	45	-67	-29	Right Cerebellum, Posterior Lobe
	19.9	-37	-75	-33	Left Cerebellum, Posterior Lobe
	16.9	3	-28	-27	Pons
	14.6	-27	-53	-18	Left Cerebellum, Anterior Lobe
	11.2	30	-43	-30	Right Cerebellum, Anterior Lobe
					
**high-dimension-warping (perfusion)**					
		**Talairach coordinate**			
			
**Cluster size**	**t value**	**x**	**y**	**z**	**Region**

25688	23.2	-49	-58	-34	Left Cerebellum, Posterior Lobe
	22.6	-2	-45	-5	Left Cerebellum, Anterior Lobe
	21.3	34	-75	-33	Right Cerebellum, Posterior Lobe
	21.2	6	-43	-8	Right Cerebellum, Anterior Lobe
	18.8	-2	-21	-24	Pons
					
**high-dimension-warping (atrophy)**					
		**Talairach coordinate**			
			
**Cluster size**	**t value**	**x**	**y**	**z**	**Region**

12947	16.1	-18	-49	-14	left cerebellum, Anterior lobe
	14.2	-4	-73	-27	left cerebellum, Posterior lobe
	12.1	22	-54	-21	Right cerebellum, Anterior lobe
	9.6	46	-72	-22	Right Cerebellum, Posterior Lobe
1416	14.2	-2	-20	-26	Pons

## Discussion

The present results of significant perfusion decreases in the cerebellum and pons of the MSA-C patients are the same as those noted in a previous report using SPM analysis of SPECT data with conventional warping [7]. In addition to perfusion data, high-dimension-warping of brain SPECT presented morphological data using a TBM technique. This high-dimension-warping achieves sufficient spatial normalization of locally atrophied areas to a template of Talairach space [5]. This sufficiency was confirmed in severely atrophied cerebellum and pons in the MSA-C patients. Insufficient spatial normalization by conventional warping resulted in a smaller cerebellum and pons than those seen with high-dimension-warping. This better anatomical precision obtained with high-dimension-warping than with conventional warping led to higher significance of the perfusion decrease in the whole cerebellum and pons of the MSA-C patients. A disadvantage of this high-dimension-warping is slight modification of the tracer activity in the original SPECT images in contrast to its preservation in conventional warping. However this modification of tracer activity remained within 3% on the average of the original tracer distribution in the present study.

A TBM technique utilizes information from high resolution deformation tensor fields obtained from the non-linear transformations of individual MRI to the template [8]. This TBM technique was originally used for investigation of longitudinal morphological changes in the same individuals using 3 D volume data of MRI [3,6]. This technique has been recently extended to cross-sectional studies for characterization of morphological changes in specific diseases [9,10]. Prior to high-dimension-warping, there is a need for skull stripping to extract only brain tissue from brain MRI [6]. Incomplete skull stripping substantially deteriorates the precision of high-dimension-warping. This deterioration gives rise to errors in Jacobian determinant values. Unlike MRI data, skull activity is almost negligible in SPECT images. This neglect of skull activity is advantageous to SPECT imaging when applying high-dimension-warping. To the best of our knowledge, this is the first report on application of TBM to brain perfusion SPECT. This technique succeeded in extracting information on inherent local atrophy from SPECT. Clinical validation could be obtained in the MSA-C patients in the present study because MSA-C manifests a characteristic pattern of atrophy in the whole cerebellum and pons on MRI [2,11]. This characteristic pattern of atrophy was clearly demonstrated by TBM of SPECT.

We must also make note of several study limitations. First, we investigated the applicability of TBM to brain perfusion SPECT in MSA-C patients with severely localized cerebellar and pontine atrophy in the present study. Further investigation on the relationship between atrophy or perfusion changes measured by the present technique and disease severity may be necessary in a larger number of patients. Moreover we have to investigate this applicability in other neurodegenerative diseases with mild atrophy. Second, comparative studies between MRI and SPECT for the evaluation of local atrophy may be necessary for additional validation of this technique. How fine the local atrophy is evaluated may depend on the anatomical precision of a SPECT template. Third, this technique cannot perform partial volume correction in brain perfusion SPECT. However additional information on local atrophy is of great aid in the interpretation of brain perfusion SPECT.

## Conclusions

The present technique of application of TBM to brain perfusion SPECT provides information on both local perfusion and atrophy using SPECT alone. The utility of this technique was clinically validated in MSA-C patients as compared to normal controls. Further study is expected on the SPECT data of individual subjects as compared to a normal database of perfusion and atrophy images for routine clinical studies.

## Competing interests

The authors declare that they have no competing interests.

## Authors' contributions

HM designed the procedure and the validation protocol of this analysis and drafted the manuscript. EI and IK co-designed the validation protocol and co-drafted the manuscript. AS, KI, YS, and NS performed data analysis in this paper. DK and MY developed a software program for the present analysis.

All authors read and approved the final manuscript.

## Pre-publication history

The pre-publication history for this paper can be accessed here:

http://www.biomedcentral.com/1471-2342/10/17/prepub
